# Elevated serum urate is a potential factor in reduction of total bilirubin: a Mendelian randomization study

**DOI:** 10.18632/oncotarget.21977

**Published:** 2017-10-24

**Authors:** Hui Zhang, Jing Liu, Zheng Dong, Yue Ding, Qiaoxia Qian, Jingru Zhou, Yanyun Ma, Zhendong Mei, Xiangxiang Chen, Yuan Li, Ziyu Yuan, Juan Zhang, Yajun Yang, Xingdong Chen, Li Jin, Hejian Zou, Xiaofeng Wang, Jiucun Wang

**Affiliations:** ^1^ State Key Laboratory of Genetic Engineering, Collaborative Innovation Center for Genetics and Development, School of Life Sciences, Fudan University, Shanghai, China; ^2^ Ministry of Education Key Laboratory of Contemporary Anthropology, School of Life Sciences, Fudan University, Shanghai, China; ^3^ Fudan-Taizhou Institute of Health Sciences, Taizhou, Jiangsu, China; ^4^ Division of Rheumatology, Huashan Hospital, Fudan University, Shanghai, China; ^5^ Institute of Rheumatology, Immunology and Allergy, Fudan University, Shanghai, China

**Keywords:** serum urate, total bilirubin, mendelian randomization study

## Abstract

**Aim:**

A Mendelian randomization study (MRS) can be linked to a “natural” randomized controlled trial in order to avoid potential bias of observational epidemiology. We aimed to study the possible association between serum urate (SU) and total bilirubin (TBIL) using MRS.

**Materials and Methods:**

An observational epidemiological study using ordinary least squares (OLS) regression and MRS using two-stage least square (TLS) regression was conducted to assess the effect of SU on TBIL. The comparison between the OLS regression and the TLS regression was analyzed by the Durbin-Hausman test. If the *p* value is significant, it suggests that the OLS regression cannot evaluate the relationship between exposure and outcome, and the TLS regression is precise; while if the *p* value is not significant, there would be no significant difference between the two regressions.

**Results:**

A total of 3,753 subjects were analyzed. In OLS regression, there was no significant association between SU and TBIL in all subjects and subgroup analysis (all *p* > 0.05). However, MRS revealed a negative correlation between SU and TBIL after adjustment for confounders (beta = –0.021, *p* = 0.010). Further analysis was conducted in different SU subgroups, and results show that elevated SU was associated with a significant reduction in TBIL after adjustment for hyperuricemic subjects (beta = –0.053, *p* = 0.027). In addition, the results using the Durbin-Hausman test further confirmed a negative effect of SU on TBIL (*p* = 0.002 and 0.010, respectively).

**Conclusions:**

This research shows for the first time that elevated SU was a potential causal factor in the reduction of TBIL and it provides strong evidence to resolve the controversial association between SU and TBIL.

## INTRODUCTION

Uric acid is the end-product of purine metabolism in humans and is controlled by several genetic factors [1], such as *ABCG2* and *SLC2A9* [2]*.* It has been reported that hyperuricemia can be one of the factors inducing oxidative stress followed by a pro-inflammatory process and endocrine dysfunction in adipose tissue [3]. Bilirubin had powerful antioxidant properties [4–6] and could be oxidized to biliverdin by reactive oxygen species (ROS) which could then protect cells from oxidative stress [7]. Observational studies about the relationship between serum urate (SU) and total bilirubin (TBIL) levels were controversial. An observational study conducted in Korea has suggested that SU positively correlates with TBIL levels [8]. However, two reports found no association between SU and TBIL in Korea and Japan [9, 10]. Surprisingly, a researcher in Serbia found that SU were positively associated with TBIL in men, but not in women [11]. It was generally known that the results from conventional observational epidemiology were more likely misinterpreted by a variety of confounders, such as lifestyle, socioeconomic factors, baseline health status, reverse causation, selected bias or other potentially unknown factors [12]. For example, SU levels were strongly affected by hormonal factors [13], body composition [14], diuretic use [15] and son on. Therefore, it was difficult to confirm whether SU was a real mediator of TBIL or whether the observed relationship between SU and TBIL was attributable to confounders. Therefore, it is difficult to confirm whether SU is a real causal of TBIL or whether the observed relationship between SU and TBIL was attributable to confounders.

The Mendelian randomization study (MRS) has been linked to a “natural” randomized controlled trial [16, 17]. It is an approach to identify genetic variants (instrumental variables) that represent the intermediate phenotype (serum uric acid in this study) and the effect of the instrument on outcome (TBIL in this study). Therefore, it can be used to study whether an intermediate phenotype has a causal effect on outcome [18]. A study with genetic variants employed as instrumental variable could avoid potential bias of observational epidemiology that comes from confounders and reverse causation [19, 20]. MRS has developed and progressed methodologically and increasingly applied in epidemiology to measure the causal relationship between exposures and outcomes in recent years. In addition, it has been gradually expanding the landscape of causal relationships in non-communicable chronic diseases [21]. In addition, the combined information of multiple variants as instruments in the study would increase statistical power to test the relationship between exposures and outcomes [22–24] and would avoid potential bias caused by weak instruments [25].

In this study, we used the MRS with genetic variants as the instrumental variable to investigate the potentially causal relationship between SU and TBIL levels.

## RESULTS

### The reliability of the instrumental variable

A total of 3,753 subjects, including 2,713 males and 1,040 females, were recruited in this study. The mean age of the population was 69.43 ± 8.96 years (Table 1). The total genetic risk score was selected as the instrumental variable in the Mendelian randomization analysis. There was a robust association between the total genetic risk score and SU; the F-statistic was 119.00. In addition, R^2^ represents the variance in SU explained by the instrumental variable, and the total genetic risk score explained 3.1% of the variance in SU in our population (Table 2). Furthermore, the other biochemical variables and the relationship between total genetic risk score and potential confounders are shown in Table 3. There was no relationship between total genetic risk score and confounders with *p* values greater than 0.05 after adjustment (age: *p* = 0.086; gender: *p* = 0.090; BMI: *p* = 0.573; FBG: *p* = 0.765; BUN: *p* = 0.765; respectively), suggesting no evidence for relationship between the total genetic risk score and other factors. Therefore, the selected instrumental variables in our MRS were adequate.

**Table 1:** Summary of the characteristics of the subjects

	All (3,753)		Males (2,713)		Females (1,040)	
	Mean	SD	Mean	SD	Mean	SD
Age (years)	69.43	8.96	70.49	8.96	66.69	8.36
BMI (kg/m2)	24.80	3.38	24.67	3.26	25.13	3.67
BUN (mmol/L)	5.65	2.10	5.78	2.29	5.33	1.44
FBG (mmol/L)	5.59	1.61	5.60	1.64	5.59	1.54
TBIL (µmol/L)	19.25	8.21	20.11	8.62	17.01	6.56
SU (µmol/L)	347.24	101.07	360.27	95.61	312.21	106.45

BMI: Body mass index; BUN: Blood urea nitrogen; FBG: Fasting blood glucose; TBIL: Total bilirubin; SU: Serum urate; SD: Standard deviation

**Table 2:** Association between uric acid transporter genetic risk score and SU

	F-statistic	R^2^	*p*
Overall	119.00	0.031	< 0.001
Males	127.50	0.045	< 0.001
Females	69.12	0.062	< 0.001

The F-statistic represents the strength between the instrumental variables and UA; R^2^ represents the variance in SU explained by the instrumental variables.

**Table 3:** Analysis of association of SU genetic risk scores with tested confounders

		beta	SE	*P*
Age				
All	Crude	-0.105	0.047	0.027
	Adjusted	-0.084	0.049	0.086
Males	Crude	-0.126	0.051	0.012
	Adjusted	-0.112	5.655	0.037
Females	Crude	0.083	0.111	0.455
	Adjusted	0.055	0.113	0.625
FBG				
All	Crude	0.194	0.263	0.459
	Adjusted	0.150	0.266	0.573
Males	Crude	-0.246	0.278	0.376
	Adjusted	-0.262	0.279	0.349
Females	Crude	1.109	0.600	0.065
	Adjusted	0.540	0.624	0.387
BUN				
All	Crude	-0.003	0.202	0.986
	Adjusted	0.061	0.203	0.765
Males	Crude	0.028	0.198	0.886
	Adjusted	0.065	0.200	0.746
Females	Crude	1.084	0.640	0.094
	Adjusted	0.965	0.655	0.141
BMI				
All	Crude	0.149	0.126	0.235
	Adjusted	0.102	0.203	0.765
Males	Crude	0.223	0.140	0.112
	Adjusted	0.182	0.143	0.199
Females	Crude	0.269	0.252	0.285
	Adjusted	0.206	0254	0.417

Each independent test variable was adjusted by the other three variables (gender has been adjusted in All group). SE: Standard error; BMI: Body mass index; BUN: Blood urea nitrogen; FBG: Fasting blood glucose; SE: Standard error.

### Elevated serum urate was a potential causal factor in reduction of TBIL

The results of conventional epidemiological analysis by the OLS method and Mendelian randomization analysis with the TLS approach are shown in Table 4. In the OLS regression, there was no significant relationship between SU and TBIL (for males: beta = -0.003; *p* = 0.061; for females: beta = 0.002, *p* = 0.201; respectively); after adjusting for age, BMI, BUN and FBG, similar results were found between SU and TBIL in gender subgroups (male: beta = -0.001, *p* = 0.589; female: beta = 0.002, *p* = 0.242; respectively). However, using the TLS approach, a negative correlation between SU and TBIL was identified (beta = -0.021, *p* = 0.009). In gender subgroup analysis, the negative correlation was significant in males (beta = -0.020, *p* = 0.035) but not in females (beta = -0.006, *p* = 0.491). Similar results were also observed after adjustment for confounders (for all: beta = -0.021, *p* = 0.010; for males: beta = -0.021, *p* = 0.037; for females: beta = -0.006, *p* = 0.491). In addition, the comparison between the OLS analysis and the TLS analysis was calculated, and a significant difference was found in the adjusted analysis (for all: DHp = 0.002; for males: DHp = 0.038; for females: DHp = 0.320). Therefore, these results suggest that elevated serum urate was a potential causal factor in reduction of TBIL.

**Table 4:** The OLS regression and the TLS regression analysis of SU against TBIL

		The OLS regression			The TLS regression			DH p
		beta	SE	*p*	beta	SE	*p*	
All (3753)	Crude	0.002	0.001	0.224	–0.021	0.008	**0.009**	**0.003**
	Adjusted	0.003	0.002	**0.025**	–0.021	0.008	**0.010**	**0.002**
Males (2713)	Crude	–0.003	0.002	0.061	–0.020	0.010	**0.035**	0.066
	Adjusted	–0.001	0.002	0.589	–0.021	0.010	**0.037**	**0.038**
Females (1070)	Crude	0.002	0.002	0.201	–0.005	0.009	0.604	0.410
	Adjusted	0.002	0.002	0.242	–0.006	0.009	0.491	0.320

SE: Standard error; DH p: Durbin-Hausman *p* value; Adjusted for age, gender (in All), BMI, BUN and FBG; DHp: Durbin-Hausman *p* value; TLS: two-stage least square; OLS: ordinary least squares.

### Elevated serum urate was a potential causal factor in reduction of TBIL in individuals with hyperuricemia

Further studies were conducted in normal and hyperuricemia subgroups (Table 5). In the normal SU subgroup, a significant association between SU and TBIL was found by the OLS regression (for crude: beta = 0.013, *p* < 0.001; for adjusted: beta = 0.011, *p* < 0.001). However, when stratified by gender, no significant association was found in either males or females (*p* > 0.05). In the TLS regression analysis, there was no significant association between SU and TBIL (for crude: beta = -0.047, *p* = 0.109; for adjusted: beta = -0.047, *p* = 0.092), and the DH*p* value was 0.025 and 0.002 in crude and adjusted analysis respectively, suggesting the result of OLS regression was affected by potential confounders or reverse causality, because it was different from the results of the TLS regression. Moreover, no association was observed between SU and TBIL in males (for crude: beta = 0.006, *p* = 0.085; for adjusted: beta = 0.007, *p* = 0.063) and females (for crude: beta = 0.018, *p* = 0.477; for adjusted: beta = 0.010, *p* = 0.698).

**Table 5:** The OLS regression and the TLS regression analysis of SU against TBIL in participants with high and normal SU

Participants		The OLS regression			The TLS regression			DH p
		beta	SE	*p*	beta	SE	*p*	
Normal SU								
All (2794)	Crude	0.013	0.003	**< 0.001**	–0.047	0.029	0.109	**0.025**
	Adjusted	0.011	0.003	**< 0.001**	–0.047	0.028	0.092	**0.002**
Males (1975)	Crude	0.006	0.004	0.085	–0.037	0.033	0.269	0.180
	Adjusted	0.007	0.004	0.063	–0.037	0.033	0.264	0.170
Females (819)	Crude	–0.001	0.004	0.849	0.018	0.025	0.477	0.450
	Adjusted	–0.003	0.004	0.415	0.010	0.045	0.698	0.590
Hyperuricemia								
All (959)	Crude	–0.003	0.004	0.441	–0.053	0.028	**0.053**	**0.045**
	Adjusted	0.001	0.004	0.854	–0.053	0.028	**0.027**	**0.010**
Males (733)	Crude	–0.007	0.005	0.141	–0.046	0.030	0.124	0.170
	Adjusted	–0.003	0.005	0.523	–0.056	0.028	**0.049**	**0.039**
Females (226)	Crude	0.007	0.007	0.297	–0.086	0.061	0.158	0.036
	Adjusted	0.010	0.007	0.147	–0.107	0.090	0.240	0.050

Adjusted for age, gender (in All), BMI, BUN and FBG; SE: Standard error; TLS: two-stage least square; OLS: ordinary least squares; DHp: Durbin-Hausman p value

In addition, the OLS and TLS regression was also performed in hyperuricemia subjects (Table 5). In the OLS regression, no relationship between SU and TBIL was found in both crude (for all: beta = -0.003, *p* = 0.441; for male: beta = -0.007, *p* = 0.141; for female: beta = -0.007, p = 0.141) and adjusted analysis (for all: beta = 0.001, *p* = 0.854; for male: beta = -0.003, *p* = 0.523; for female: beta = 0.010, *p* = 0.147). In the TLS regression analysis, there was no significant relationship between SU and TBIL in crude analysis (beta = -0.053, *p* = 0.053), but after adjusting for confounders, increased SU was associated with significant reduction of TBIL in hyperuricemia individuals (beta = -0.053, *p* = 0.027); the Durbin-Hausman test provided further evidence of a negative effect of SU on TBIL (DHp = 0.010). Similarly, further analysis was conducted in gender subgroups, and significant association was found in males (beta = -0.056, *p* = 0.049) but not in females (beta = -0.107, *p* = 0.240) after adjustment for age, BMI, BUN and FBG. The Durbin-Hausman test provided further evidence of a negative effect of SU on TBIL (DHp = 0.039 and 0.050, respectively).

## DISCUSSION

The theoretical foundation of Mendelian randomization depends on three assumptions [12, 26, 27]. (I) The instrumental variable is robustly associated with modifiable exposure of interest, with the F-statistic being considerably greater than ten [28]. In this study, the F-statistic was 119.00 and the R^2^ was 0.031 (Table 2), indicating a robust relationship between the genetic risk score (instrumental variable) and SU (exposure of interest). (II) The instrumental variables are not associated with confounding factors that cause biased conventional epidemiological associations between modifiable risk factors and outcomes. This assumption could be examined by verifying the instrumental variable for association by multivariate linear regression with adjusted for confounders (Table 3). There was no evidence for a relationship between the uric acid transporter as the instrumental variable and any other confounders tested, all *p* values were greater than 0.05 (age, gender, BMI, FBG and BUN). (III) The instrumental variables are related to the outcome only via its association with the modifiable exposure. This assumption was difficult to assess, and should be considered Despite this, the possibility of pleiotropic effects of the uric transporter instrumental variable on TBIL levels was difficult to exclude [29]. We did note that some of the uric acid transporters (particularly *ABCG2* [30]) were widely expressed and may have pleiotropic effects (i.e. effects on TBIL levels aside from or in addition to a direct effect of the serum urate). To reduce the possibility of violating the assumption, the Mendelian randomization approach was adjusted by age, gender, BMI, FBG and BUN in our study. Therefore, we were able to show that the selected instrumental variables in our MRS were adequate.

This study investigated for the first time the association between SU and TBIL levels using MRS. The Mendelian randomization employed the TLS approach with the uric acid transporter risk score as the instrumental variable; the results provided evidence that the SU levels were negatively correlated with TBIL. The Durbin-Hausman test provided further proof of the negative effect between exposure (SU) and outcomes (TBIL) with significant differences (DHp = 0.002) in the adjusted analysis of TBIL. In addition, the individual SNPs were also analyzed in our study (Table 6). At *ABCG2* (rs1481012), negative beta and significant DH*p* values (beta = -0.019, *p* = 0.024, DHp = 0.006) were observed, suggesting the best evidence for a causal association between elevated SU and reduction of TBIL in this study. We also noted, from the F-statistics and R^2^, that *ABCG2* (rs2231137), *SLC2A9* (rs16890979) and *SLC17A1* (rs3799352), were weak instrumental variable and contributed very little to this analysis.

**Table 6:** The TLS regression analysis of SU against TBIL using a single genetic variant as the instrument

Gene (SNP)	Subjects	F-statistic	R^2^	beta	SE	*p*	DH p
*ABCG2* (rs1481012)	All	107.50	0.028	–0.019	0.008	**0.024**	**0.006**
	Males	78.33	0.028	–0.026	0.011	**0.019**	**0.017**
	Females	46.67	0.043	0.003	0.009	0.779	0.990
*SLC2A9* (rs16890979)	All	10.13	0.003	–0.133	0.058	**0.023**	**< 0.001**
	Males	5.72	0.002	–0.186	0.112	0.097	**< 0.001**
	Females	9.05	0.009	–0.046	0.029	0.109	**0.036**
*ABCG2* (rs2231137)	All	35.51	0.009	–0.010	0.017	0.429	0.300
	Males	34.91	0.013	–0.006	0.015	0.695	0.740
	Females	5.75	0.006	–0.028	0.033	0.388	0.300
*SLC17A1* (rs3799352)	All	7.99	0.002	–0.004	0.031	0.900	0.980
	Males	7.48	0.003	0.018	0.035	0.609	0.580
	Females	1.24	0.001	–0.056	0.101	0.576	0.440

Adjusted for age, gender (in All), BMI, BUN and FBG; DHp: Durbin-Hausman *p* value; TLS: two-stage least square.

The assumption that elevated SU levels decrease the TBIL levels via induction of ROS and insulin resistance was considered. Tentative evidence was observed to support this hypothesis in previously published studies. Increasing concentrations of SU levels in the culture media of adipocytes induced a further increase in intracellular ROS production with activated NADPH [3]; others have reported that SU induces oxidative stress in adipocytes, vascular smooth muscle cells and human umbilical vein endothelial cell [31, 32]; Zhu et al. recently reported that hyperuricemia could increase ROS levels [33]. However, bilirubin showed powerful antioxidant properties [4–6] and could be oxidized to biliverbin by ROS, which can then protect cells from oxidative stress [7]. On the side, several studies has reported that high uric acid inhibits insulin signaling and induces insulin resistance [33, 34]. Interestingly, bilirubin could increase insulin sensitivity in published reported [35, 36]. Taken together, these studies support the hypothesis that elevated SU levels can lead to increased ROS levels and induced insulin resistance, and resulting in reduction of TBIL levels. In addition, elevated SU may increase ROS levels in females, and 17β-estradiol might inhibit expression of ROS [37–39]; therefore, the relationship between SU and TBIL was not significant in females. However, we found that the association between SU and TBIL is significant in males. Although the mechanism that underlies this gender-specific association is unclear, in this and other studies, higher levels of urate and bilirubin were observed in males than females [40, 41], which may explain the different statistical power of detection of urate-bilirubin associations in males vs. females. Our study provides an important insight in the understanding the potential mechanism of TBIL, elevated serum urate is a potential factor in reduction of total bilirubin. In addition, several published studies have been reported that bilirubin was a decrease risk factor in diabetes mellitus [42, 43] and cardiovascular disease [44, 45]. Thus, it was quite important for physician to emphasize the risk of elevated SU and subjects should be strongly recommended to decrease high level of SU to avoid diabetes mellitus and cardiovascular disease.

Our study has a number of strengths. First, we used both the OLS and the Mendelian randomization approach, not merely a conventional observational method, to investigate exposure-outcome relationships and the potential relationship between SU and TBIL-related phenotypes in a Chinese population. This method allows a more precise conclusion of causality compared with traditional observational studies [19]. Second, the instrumental variable consisted of four SNPs, and the combined use of multiple variants as instruments increased the statistical power to test relationships between exposure and outcomes [22–24] and avoided the bias caused by weak instruments [25]. Finally, the uric acid transporter SNPs used were sufficient to represent the hereditary background of the Chinese population [46]. Nevertheless, this analysis has several limitations. Our study was conducted only with an older population, and further studies should be conducted in the general population. The data we analyzed was from a single, relatively small cohort, and further confirmation on our results in other cohorts from the Chinese population would be necessary. And our research merely focused on Chinese subjects, and studies using Mendelian randomization approach should be repeated in other populations.

In summary, although the relationship between SU and TBIL was likely to be very complex, our findings provide strong evidence that elevated SU levels is a potential factor in reduction of TBIL. To the best of our knowledge, this is the first research that investigates the relation between SU and TBIL using Mendelian randomization analysis, and it generated strong evidence to resolve the controversial association between SU and TBIL. Furthermore, our study provides an important insight in the understanding the potential mechanism of TBIL.

## MATERIALS AND METHODS

### Study population

The study population and methods of data collection were described previously [46]. This present study included 3,753 subjects (2,713 males and 1,040 females), and subjects who have been treated with urate-lowering drugs were excluded from this study. Characteristics of the participants, including age, gender, body mass index (BMI), fasting blood glucose (FBG), blood urea nitrogen (BUN), total biliverdin (TBIL) and serum urate (SU), were analyzed (Table 1). Subjects with high serum urate (> 417 µmol/L) were considered hyperuricemia patients [47]. This study was approved by the Ethical Committees of the School of Life Sciences of Fudan University and informed consent was obtained from each participant.

### Instrumental variables

For the uric acid transporter genetic risk score, SU-increasing alleles were selected based on our previous report [46]. In that study, in order to find the serum urate associated genes, 31 loci were tested in all individuals in Chinese. Then 11 loci in eight genes were shown to be associated with SU after multiple corrections. In summary, four single nucleotide polymorphisms (SNPs), which were reported to be associated with SU in literature, were selected as instrumental variables in our study: rs1481012 in *ABCG2* (beta = 27.555, *p* = 3.50E-31, P_FDR_ = 1.02E-29)*,* rs2231137 in *ABCG2* (beta = -16.945, *p* = 2.33E-12, P_FDR_ = 3.38E-11), rs16890979 in *SLC2A9* (beta = -24.654, *p* = 0.014, P_FDR_ = 0.042) and rs3799352 in *SLC17A1* (beta = -7.456, *p* = 5.35E-3, P_FDR_ = 0.017). We selected those four loci based on the following consideration: 1) The loci explain 4.2% of the variance of SU concentration, while approximately 7% for all known loci. 2) The loci have strong and unequivocal contributions to the concentration of SU based on our lab’s previous study [46] and the reported genome-wide association studies [48–50]. However, regarding other loci, they show weak and ambiguous effects on SU and may cause controversial results. 3) Ethnicity have been proved to be a heterogeneous source for the association between genetic loci and SU. Some loci identified in EU and US populations did not affect serum urate levels in Chinese populations [51]. In our previous study, we had systematically studied the genetic basis of SU with all potential urate-related SNPs and found those four loci significantly influenced the concentrations of serum urate in this population [46]. 4) The loci corresponding genes encode urate transporters, which directly modify the SU concentrations. In order to get a more precise and powerful genetic risk score to explain the variance of SU, a weighted genetic risk score was calculated. An allele-counting genetic risk score was calculated. Based on the number of minor alleles that were associated with increased SU levels, each SNP was coded 0–2, and that number was multiplied by the corresponding beta value, and then the total genetic risk score was determined as the sum the four values. Most importantly, the instrumental variables selected must fulfill three assumptions [12, 26, 27]: the instrumental variables should be (1) robustly associated with the modifiable exposure of interest; (2) independent from confounding factors; (3) related to the outcome only via their association with the modifiable exposure.

### Statistical analysis

First, the traditional observed relationship between SU and TBIL levels was analyzed using the ordinary least squares (OLS) regression. Second, the MRS was determined by conducting a two-stage least square (TLS) regression as previously described [52]. As shown above, the total genetic risk score calculated by four SNPs was selected as the instrumental variable in the TLS regression to assess the relationship between SU and TBIL levels. In the MRS, the F-statistic was examined from the first-stage regression and represents the strength between instrumental variables and exposures [53]; R^2^ represents the variance in SU as explained by the instrumental variables. Both the F-statistic and determination coefficient (R^2^) were measured. According to the assumption of the Mendelian randomization approach, the F-statistic should be greater than ten [28], which would suggest that genetic instrumental variables were powerful enough to represent the level of SU. The relationship between the total genetic risk score and potential confounders was also assessed by linear regression. For the last step, the difference between the OLS regression and the TLS regression was measured by the Durbin-Hausman test [54]. If the *p* value is significant, it suggests that the OLS regression cannot evaluate the relationship between exposure and outcome, and then the TLS regression is used for further analysis; while when the *p* value is not significant, both the OLS and TLS regression can be used.

All analyses were carried by R (Version 3.2.2: www.r-project.org/). *P* value less than 0.05 was determined statistical significance.
